# What Is the Smallest Change in Pulse Wave Velocity Measurements That Can Be Attributed to Clinical Changes in Arterial Stiffness with Certainty: A Randomized Cross-Over Study

**DOI:** 10.3390/jcdd10020044

**Published:** 2023-01-25

**Authors:** Mario Podrug, Borna Šunjić, Pjero Koren, Varja Đogaš, Ivana Mudnić, Mladen Boban, Ana Jerončić

**Affiliations:** 1 Laboratory of Vascular Aging, University of Split School of Medicine, 21000 Split, Croatia; 2 Department of Health Studies, University of Split, 21000 Split, Croatia; 3 Department of Psychological Medicine, University of Split School of Medicine, 21000 Split, Croatia; 4 Department of Basic and Clinical Pharmacology, University of Split School of Medicine, 21000 Split, Croatia; 5 Department of Research in Biomedicine and Health, University of Split School of Medicine, 21000 Split, Croatia

**Keywords:** arterial stiffness, pulse wave velocity, reproducibility, repeatability, minimal clinically important difference, MCID, intra-subject variability, measurement errors

## Abstract

Pulse wave velocity (PWV), a direct measure of arterial stiffness, is a promising biomarker of cardiovascular risk and a cardiovascular surrogate outcome. The resolution for detecting its smallest clinically significant change is dependent on the expected reproducibility, but there is currently no consensus on this. We estimated the PWV reproducibility in a range of intra-subject values that were observed over a 2 week period in a broad range of participants and under clinically relevant experimental conditions (two observers, morning/afternoon sessions, and number of visits) using SphygmoCor and Arteriograph devices. Each participant was recorded 12 times with each device over three visits, one week apart, and two morning and two afternoon recordings were taken per visit. The factors affecting reproducibility and the discrepancies between the consecutive PWV measurements for each device were also examined using multilevel mixed-effect models. We show that current PWV estimation guidance recommending 2 + 1 measurements is suboptimal because the PWV range was outside of the 1 m/s threshold for most of the participants, which is proposed as a minimal clinically important difference. The best reproducibility was yielded with median of four measurements and a 1.1 m/s threshold. Although PWV reproducibility and repeatability are frequently used interchangeably in studies, we demonstrated that despite their relative measures of variability (e.g., coefficient of variation) being comparable, their ranges revealed a clinically significant difference between them. We also found that different physiological variables were predictors of the discrepancy between the consecutive measurements made by the two devices, which is likely due to their distinct modes of operation. The evidence base for PWV reproducibility is limited, and more research is needed to deepen our understanding of the variation in arterial stiffness over time, as well as fluctuations within a population group and in an intervention setting.

## 1. Introduction

Arterial stiffening is the most characteristic clinical feature of the aging process in the arterial system, which is characterized by a decrease in the arterial compliance and/or changes in the arterial wall characteristics [1,2]. Numerous studies performed on both patient- and population-based samples have found that more arterial stiffness is independently associated with an increased risk of having a first or recurrent major cardiovascular disease event [3,4,5]. Because the measurement of aortic stiffness is viewed as an integrator of all of the damage that has been incurred in previous years to the arterial wall in response to both traditional and poorly identified/unidentified cardiovascular risk factors, arterial stiffness is considered to be a good biomarker for the detection of early vascular aging [2,6,7,8], as well as a surrogate endpoint for cardiovascular disease [9,10]. Measurements of arterial stiffness have been shown to improve the reclassification of patients who are at intermediate risk for cardiovascular disease by supplementing the information provided by the traditional risk factors [3,11,12,13]. Additionally, it has been demonstrated that arterial stiffness is associated with target organ damage [14,15,16,17]. Recently, clinical trials evaluating arterial stiffness as a surrogate endpoint for cardiovascular disease in hypertensive patients have begun [18,19].

In a 2006 consensus document, the measurement of carotid–femoral pulse wave velocity (PWV) was defined as the gold standard for measuring arterial stiffness [20].

Despite their significant potential for cardiovascular disease prevention and hypertensive treatment management, the use of PWV measurements in clinical practice is limited. One of the barriers impeding PWV translation to clinical practice is a lack of methodological consensus, which hampers accurate comparisons of PWV within and between studies. The number of consecutive measurements used in the estimation of the true PWV value is an example of such an inconsistency [21]. To estimate a true PWV value, the American Heart Association (AHA) recommends averaging at least two PWV measurements, and if their difference exceeds 0.5 m/s, a third measurement should be taken, and the median value should be reported [22]. Nonetheless, some researchers employ a measurement protocol that entails repeating the measurements until two values are within 0.5 m/s of each other [23,24,25,26]. Furthermore, duplicate measurements are averaged in many studies regardless of their difference [27,28,29,30], while some studies use a single PWV measurement [31,32]. Despite its significance, the AHA recommendation is based on a single study [33], and it is classified as having a weak evidence [22]. No study, so far, has evaluated the effect of the number of measurements used in PWV estimation on the reproducibility of the PWV.

Another issue with translating PWV measurements to clinical practice is a lack of consensus on the expected reproducibility of PWV, which is defined as the precision of the measurements obtained under different conditions over a short period, typically days or weeks. Such a quantification is required to correctly interpret the results of longitudinal studies monitoring PWV changes in an individual over time and, consequently, detect the minimal clinically important change. However, currently, the reproducibility of PWV appears to have mainly been investigated in validation studies comparing new, to the reference device [34]. Only a few studies that investigated the PWV reproducibility of a single device typically used small sample sizes (N ≤ 21) and reported reproducibility as the coefficient of variation (CV), which is a relative measure expressed that is in units of standard deviation and is difficult to interpret when one is looking for clinically relevant changes or the correlation coefficient, e.g., interclass correlation coefficient [35,36]. Some of these studies additionally reported repeatability, which they expressed as the mean difference between two PWV measurements and corresponding limits of agreements [36], whereas others reported repeatability as a precision of measurements obtained under the same conditions within 24 h, rather than reproducibility [33,37,38,39,40]. To assess PWV reproducibility, the variability that occurs due to different experimental settings and random factors other than clinically relevant change, powered studies on a specific device are required, with PWV reproducibility being expressed in a measurement unit that is easily interpretable in the context of determining the minimal clinically relevant change, and with PWV measurements spanning more than two measurements that are separated by a longer period than 1–2 days.

The goal of this study was to determine the amount of intra-subject PWV variability that could be attributed to conditions/factors other than the clinically relevant change by monitoring the PWV over 2 weeks in a broad range of participants and under different experimental conditions resembling those in clinical practice (different observers, time of day, and number of visits). The resolution for detecting the smallest clinically significant change is determined by this value. We also wanted to see how the number of repeated measurements used in PWV estimation affected the PWV reproducibility and what factors contributed to discrepancies between the consecutive PWV measurements. The analyses were performed separately for the two devices that use different PWV measurement techniques: the applanation tonometry device SphygmoCor CVMs, a gold standard device for PWV measurement, and the Arteriograph, an oscillometric device.

## 2. Methods

The methods for this study are detailed in another study in which we investigated the factors influencing PWV measurements and measurement difficulties [41], and they are summarized herein.

### 2.1. Participants

This 2 week long longitudinal study enrolled 36 participants between 20 and 60 years of age. The participants were evenly distributed by age (in decades), sex, hypertension status (normotensive or hypertensive), and body mass index (BMI) (normal weight, overweight, or obese). All of the invited participants provided their medical history, and those with arrhythmias, cerebrovascular sickness, pregnancy, surgery amputation, oncology disease, psychiatric disease, infections throughout the trial duration, and medical nonadherence were excluded from the study.

The Ethics Committee at the University of Split School of Medicine approved the study, and all of the participants provided written informed consent.

### 2.2. Study Design

This is a single-blind randomized cross-over longitudinal study that was conducted at the University of Split School of Medicine’s Laboratory for Vascular Aging.

**Figure 1:**
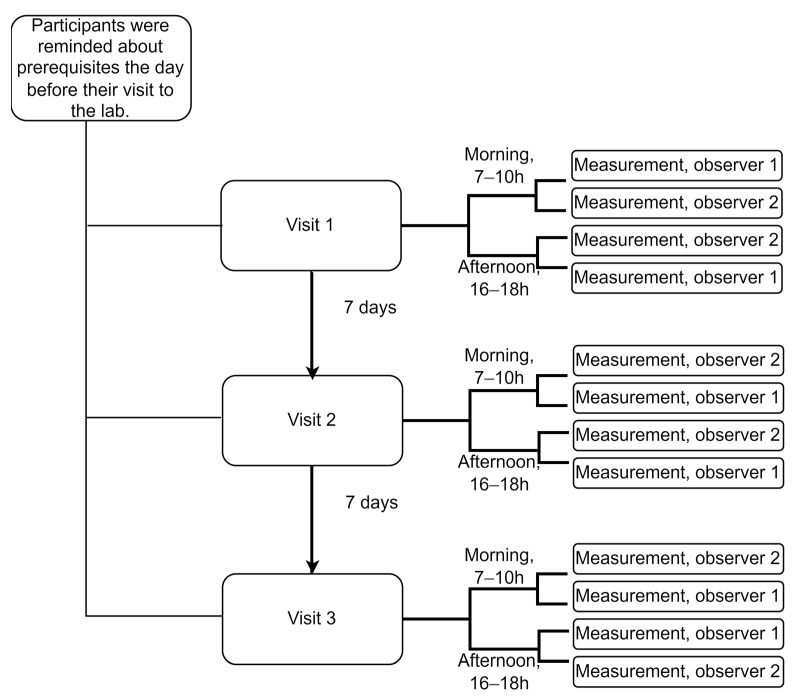
Overall study design showing recordings with a single device. The order of the observers was randomized, and their ID numbers are provided here just for illustration purposes. Prerequisites: participants were reminded to refrain from strenuous exercise and alcohol for 24 h, as well as from eating, drinking (except water), or smoking for at least 3 h before each recording session.

Over two weeks, each participant was recorded 12 times in total, four times on each of the three visit days, which were separated by one week. The two observers recorded a participant in the morning (7–10 h) and afternoon on each visit day (16–18 h) (Figure 1).

To ensure that the measurements were taken under comparable conditions by two observers, we randomized the order of the observers who were also blinded to each other’s readings. Similarly, we randomized the order of devices to ensure that the Sphygmocor CvMs and Arteriograph devices were used under the same conditions. To do so, we used randomization with a permutated block size of four and a random number generator algorithm to generate a random sequence of blocks.

### 2.3. PWV Measurements

The pulse wave measurements were taken separately using two different measuring devices: the applanation tonometer SphygmoCor CvMS (AtCor, Australia) and the oscillometric device Arteriograph (Tesniomed, Hungary). The SphygmoCor device measured the PWV between the carotid and femoral sites, and the measurements are thus referred to as carotid–femoral PWV or cfPWV in the following text. The Arteriograph, on the other hand, estimated the aortic PWV from a single site at the brachial vascular bed, which is thought to accurately approximate the cfPWV. We refer to the Arteriograph measurements in the following text as PWVao.

For both of the devices, the measurements were taken following the American Heart Association’s (AHA) recommendations for improving and standardizing the vascular research on arterial stiffness [22].

The observers performed the measurements in a quiet, temperature-controlled room at a comfortable temperature of 21–23 °C. The participants were asked to refrain from strenuous exercise and alcohol for 24 h before the recording was taken. They were also told not to eat or drink anything other than water for at least 3 h before the recording and not to smoke. Those taking vasoactive medicines were advised to continue taking them as usual and not to change the dosage during the study. To ensure hemodynamic stability, the participants rested in the supine position for 10 min before the first PWV measurement. Following the completion of the series of measurements using one device, the participants were asked to stand up, walk around the room, and then lie down for another 10 min to prepare for measurements using the second device. This step was required to keep the participants from falling asleep while lying supine for an extended period, especially in the morning. The participants were not allowed to talk or sleep during the measurements. All of the measurements were taken on the right hand (Arteriograph), and the right carotid and femoral arteries (SphygmoCor).

Before the start of the study, both of the observers had received extensive training for 7 days, during which they performed approximately 50 high-quality measurements under supervision.

To calibrate the pulse wave signals acquired using the SphygmoCor, we obtained brachial blood pressure measurements using a validated oscillometric sphygmomanometer (Welch Allyn Connex ProBP 3400 digital blood pressure monitor with SureBP technology).

To calculate the wave travel distance, we used the subtracted distance method. The method was chosen over the direct method as per the AHA guideline, the most recent guideline on arterial stiffness measurements [22]. Additionally, as per the AHA guideline, instead of using a tape measure, we used a large school divider to measure the distance between the sternal notch and the femoral measurement site, and then, we subtracted the distance between the carotid measurement site and the sternal notch. The distance between the carotid and femoral sites was only measured during the first recording session.

### 2.4. Meteorological Conditions

To describe the meteorological (outdoor) conditions under which the measurements were taken, we obtained data of the outdoor temperature, air pressure, and humidity from the Meteorological and Hydrological Service of Croatia’s local office, and we used them to estimate the weather conditions during each recording session. Throughout the study, the temperature ranged from 4.5 to 23.3 °C, the air pressure ranged from 972 to 1011 hPa, and the humidity ranged from 32 to 92%.

### 2.5. Sample Size Consideration

Assuming that we obtained a level of confidence of 95%, a population standard deviation of the intra-subject PWV changes measured using the Arteriograph of 0.57 m/s, and a margin of error of 0.2 m/s, the study would require a minimum sample size of 35 to achieve the envisaged level of precision [42]. This sample size is also sufficient to produce a level of confidence of 95% for the SphygmoCor measurements too.

### 2.6. Definitions of PWV Repeatability and Reproducibility

The AHA guideline defines the variability between the intra-subject PWV measurements separated by at least 24 h as reproducibility, which is a precision of measurements obtained under different conditions over a short period, usually days or weeks [22]. Consequently, the variability of measurements taken within 24 h and recorded under same the conditions is defined as repeatability, which isa precision of measurements obtained under the same conditions within 24 h. Usually, the repeatability and reproducibility of PWV measurements are expressed as relative measures: coefficient of variation (CV), which is reported in units of standard deviation; or correlation coefficients, such as the intraclass correlation coefficient (ICC). In this study, we express the variability as a range in m/s, which is easily interpretable in a clinical setting.

### 2.7. Data Analysis

We used descriptive statistics to describe the distribution of quantitative (mean and standard deviation or median and interquartile range (IQR), depending on the shape of distribution) and qualitative (absolute and relative frequencies) variables.

Multilevel regression models were used to account for repeated PWV measurements while identifying the factors associated with the per person count of discordant pairs of consecutive PWV measurements, the size of a discordance, or an occurrence of a discordant pair of measurements. As per the AHA guidelines, a discordant pair of PWV measurements is one where the values are more than 0.5 m/s apart [22]. Depending on the type of a dependent variable, we used multilevel mixed-effects generalized linear models for models where the size of the PWV discordance was the dependent variable, multilevel mixed-effects logistic regression models for a dichotomous dependent variable such as an occurrence of a discordant pair of consecutive PWV measurements, and multilevel mixed-effect Poisson regression for count data such as the per person count of discordant pairs of consecutive PWV measurements. All of the models used the robust estimator, which is robust to certain types of misspecification in multilevel models [43,44]. The sensitivity analysis was performed with the maximum likelihood (ML) method, without the robust estimator.

Each multiple regression model (the model including several independent variables (IVs)) was built in two steps. The experimental conditions’ variables: the order of the visit and the time of day; the meteorological (outdoor) conditions’ variables: the temperature (°C), air pressure (Pa), and humidity (%); physiological variables: the blood pressure or heart rate; participants’ characteristics: age, sex, BMI, and hypertension status, were all investigated for their relationship to the dependent variables by a simple regression analysis. Those IVs that were associated with the dependent variables at the *p* < 0.2 significance level were entered into a multiple regression model. For the IVs that were nonsignificant in a multiple model, the contribution of an IV to the model (pseudo R^2^, log pseudolikelihood, and random variance) was investigated further to decide on the final model.

## 3. Results

The study enrolled a total of 36 participants, and one participant was later removed from the study due to an ongoing infection, leaving a total of 35 participants.

We observed a wide range of PWV values (median 6.3, range 4.5–10.8 m/s, as measured using the SphygmoCor), brachial blood pressures (systolic: 126, 98–177 mmHg; diastolic: 72, 53–98 mmHg), age (41, 20–60 years), and BMI (27.3, 19.4–38.9) in our sample. In addition, the participants were distributed evenly across age (in decades), sex (17 or 49% females), hypertensive status (17 or 49% hypertensives), and BMI categories (12 or 34% normal, 11 or 32% overweight, 12 or 34% obese patients), (*p* ≥ 0.692 for all of them).

Overall, the CVs for the within-subject variation were 9.9% (95% confidence interval (95% CI) 1–19%) for the SphygmoCor and 5.3% (95% CI 0.4–10%) for the Arteriograph.

### 3.1. An Examination of Large Differences in Consecutive PWV Measurements (>0.5 m/s), for Which the AHA Recommends the Inclusion of a Third Measurement in PWV Estimation

#### 3.1.1. The Requirement for a Third Measurement

The prevalence of two consecutive PWV measurements taken within 1 h, which were more than 0.5 m/s apart, was high for the Sphygmocor device (51%, 95% CI 45–58%), and it was lower, but not insignificant, for the Arteriograph (27%, 95% CI 21–33%). In fact, the odds ratio (OR) of observing a pair of measurements with discrepancies of greater than 0.5 m/s, was 3 times higher (95% CI 2.0 to 4.6; mixed-effects logistic regression, *p* < 0.001) for SphygmoCor than for Arteriograph.

#### 3.1.2. Do Pairs of PWV Measurements with Unacceptable Large Differences Cluster within Specific Individuals?

**Figure 2:**
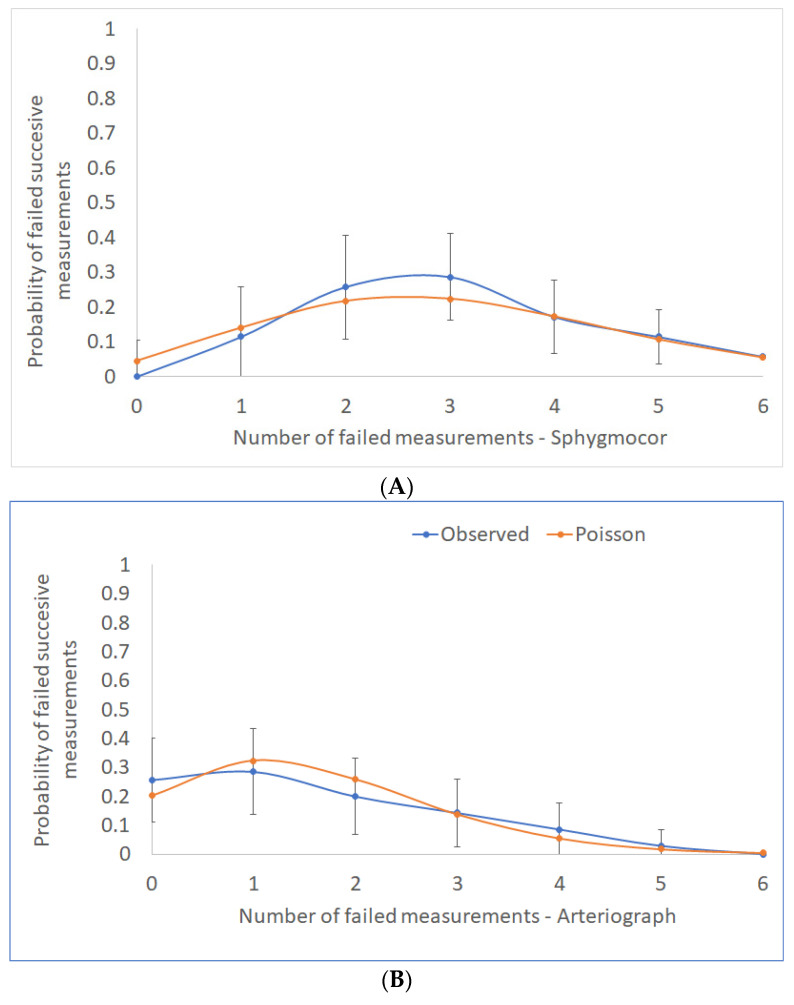
The distribution of counts per person of consecutive PWV measurements with discrepancies >0.5 m/s is shown here with the associated 95% confidence intervals (blue line). The orange line represents the theoretical Poisson distribution, which depicts discordant pairs of measurements occurring at random and independently: (**A**) SphygmoCor and (**B**) Arteriograph devices.

Out of six pairs of consecutive PWV measurements that were recorded per person, the median number of discordant pairs with a difference of greater than 0.5 m/s was three (range, 1–6) for SphygmoCor and one (0–5) for Arteriograph. However, the counts of the discordant pairs of PWV measurements per person did not deviate significantly from chance (Figure 2), implying that large differences emerge at random and independently of each other. Indeed, for the Arteriograph PWVao measurements, we found no relationship between the counts of the discordant pairs of measurements per participant and the participant’s characteristics such as age, gender, BMI, hypertension status, or the median values of the participants’ HR or BPs (simple Poisson regressions, *p* ≥ 0.074 for all of them). For the cfPWV discrepancies, these counts were weakly positively correlated to a person’s median mean arterial pressure (MAP), (simple Poisson regression, incidence rate ratio 1.017, 95% CI 1.003–1.031, *p* = 0.014) and median diastolic blood pressure (DBP) (incidence rate ratio 1.018, 95% CI 1.002–1.033, *p* = 0.027), with the models describing only up to 2% of the variation, and no other factors having been identified as predictors (*p* ≥ 0.299).

#### 3.1.3. Factors Affecting an Occurrence of a Pair of PWV Measurements with Unacceptable Large Differences

Next, we examined if any of the experimental conditions (order of visit: one, two, or three; or time of day: morning or afternoon), outdoor conditions (outdoor temperature, air pressure, or humidity) or characteristics of participants (age, gender, BMI, hypertension status, heart rate (HR) or blood pressures (BPs): MAP, systolic blood pressure (SBP) and DBP) could predict an occurrence of a discordant pair of PWV measurements with a difference of greater than >0.5 m/s. We found that increasing the median MAP and DBP values increased the odds of a discordant pair of SphygmoCor readings (simple mixed-effects logistic regressions, *p* ≤ 0.025 for both of them), while the other factors were not identified as predictors (*p* ≥ 0.263 for all of them). A one mmHg increase in the MAP and DBP raised the odds by 4% (95% CI 1–7%) and 4% (0.04–7%), respectively. In terms of the Arteriograph measurements, a one bpm increase in the median HR increased the odds of failed measurements by 7% (95% CI 1.03–1.12, *p* = 0.002), but the other factors were not significant predictors (*p* ≥ 0.252 for all of them).

### 3.2. Factors Affecting the Size of Differences between Consecutive PWV Measurements

To find out if the characteristics of the participants, experimental conditions, or outdoor conditions affected the size of the differences between the pairs of measurements, we built mixed-effects ML regression models.

We discovered that age, median MAP, but also outdoor temperature, and the interaction between the outdoor temperature and the MAP predicted the size of the discrepancies between two consecutive cfPWV measurements (Table 1). As for the PWVao discrepancies, while experimental and outdoor conditions did not affect these discrepancies (*p* ≥ 0.229 for all of them), the patients’ characteristics such as BMI, sex, or hypertension status did. The ICC for the SphygmoCor and Arteriograph revealed that the discrepancies in the consecutive PWV measurements were not well correlated within a person, for both the Sphygmocor (no correlation) and the Arteriograph (poorly correlated).

### 3.3. The Effect of the Number of Consecutive Measurements Used in PWV Estimation on 2 Weeks Reproducibility of PWV Measurements

**Figure 3:**
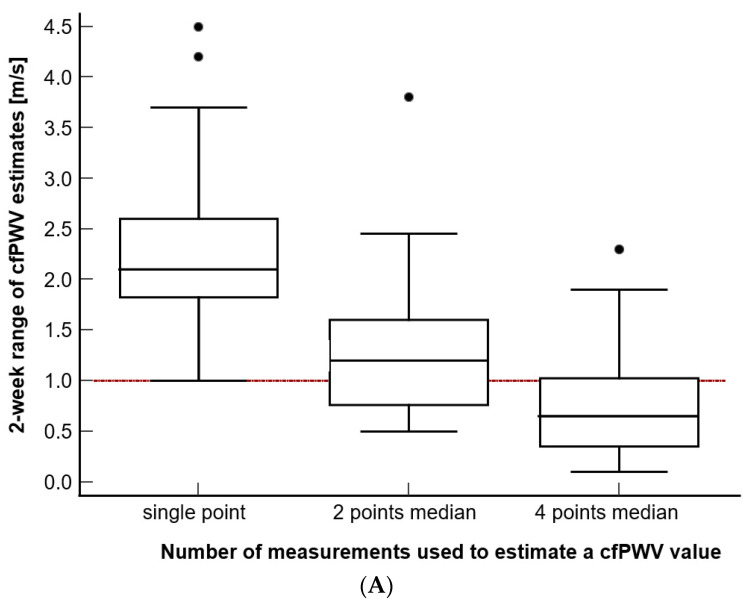

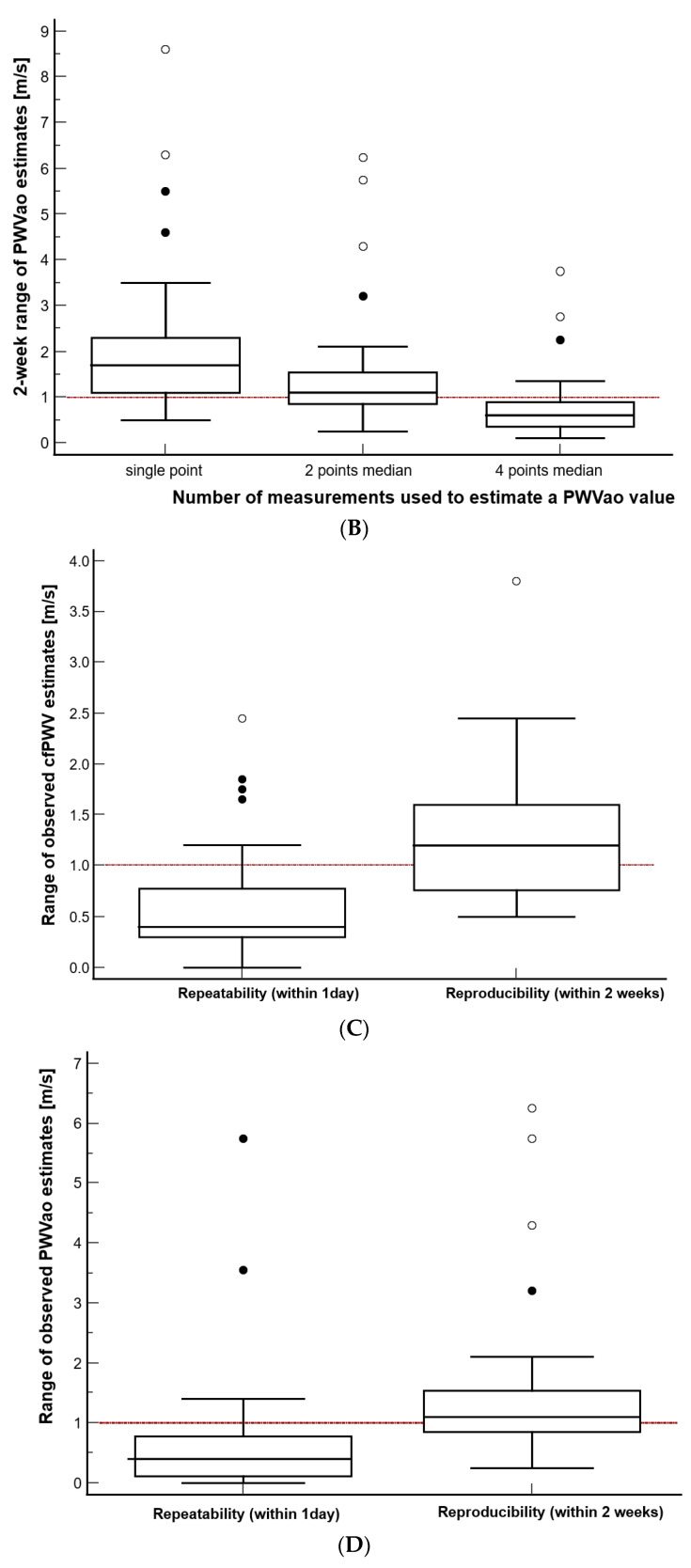
(**A**–**B**) The effect of estimating PWV from the different number of measurements that were recorded within a day. (**C**–**D**) Comparison of repeatability and reproducibility range estimated using the currently recommended strategy. The reference line at 1 m/s is noted. Additionally, please note that the value marked as 2 points median is equivalent to 2 points mean.

To investigate the effect of the number of consecutive measurements utilized to estimate the daily PWV value on the variability of PWV values observed in an individual over 2 weeks, we compared the 2 week ranges of the intra-subject PWV values when these values were estimated from a single, two (recorded within 1 h), or four consecutive measurements (recorded within 24 h) (Figure 3). We found that the reproducibility of the PWV estimates significantly decreased with the increasing number of measurements used in the estimation for both the cfPWV and PWVao values (Figure 3A and 3B, Friedman test, *p* < 0.001 for both of the measures and all of the comparisons), with the median of four consecutive daily measurements yielding the best results. Even after fully implementing the AHA recommendation for two points median/mean (two measurements plus a third measurement included in the calculation if two measurements were more than 0.5 m/s apart), the results still showed that the range for two point median PWV estimates for both the cfPWV and PWVao was greater than 1 m/s in 19 or 54% of participants. For the three points median, which is not presented in Figure 3, the respective percentages were fourteen or 40% of the participants for cfPWV and six or 17% with PWVao. On the contrary, by implementing the four points median approach, 26 participants (74%) for cfPWV and 30 of them (86%) for PWVao had their 2 week PWV values within ≤1 m/s range, whereas 30 participants (86%) for cfPWV and 31 (89%) for PWVao had 2 weeks PWV values that were within the ≤1.1 m/s range.

### 3.4. Comparison of the Reproducibility and Repeatability of PWV Measurements, When Expressed as a Range of Values

Using the currently accepted strategy of PWV estimation (mean or median of the two consecutive measurements), we then compared the repeatability of the PWV values observed in the same participant within a day to their reproducibility that was observed over two weeks (Figure 3C,D). We found that the reproducibility, when it was expressed as a range of observed values, was significantly higher for both of the PWV measures than the repeatability range was (Wilcoxon test for paired samples, *p* < 0.001 for both of the PWV measures). Specifically, the reproducibility range of cfPWV was higher by a median of 0.70 m/s (95% CI 0.55–0.87) and that of the PWVao was higher by a median of 0.68 m/s (95% CI 0.50–0.88) than their respective repeatability ranges. The repeatability, which is expressed as a range of 1 day PWV estimates, fell out of the ≤1 m/s range in eleven (10%, cfPWV) and nine (9%, PWVao) cases out of one hundred and five 1 day recording sessions, whereas the reproducibility fell out of this range in twenty-one (60%, cfPWV or PWVao) out of thirty-five participants.

### 3.5. Is There a Difference in the Size of Discrepancies between PWV Measurements Taken on the Same Day up to One Hour Apart and Those Taken on the Same Day, but Over a Longer Period?

To see if the PWV repeatability definition of the measurements needing to be taken within 24 h is supported by the data, we compared the discrepancies between the pairs of measurements taken within the same day up to 1 h apart and those taken over longer periods (8 and more hours apart), and we found no difference: mean discrepancy: 0.04 m/s, 95% CI—from 0.21 to 0.14, *p* = 0.663.

## 4. Discussion

With as many as 12 measurements per participant having been recorded over two weeks in a wide variety of experimental settings and weather conditions and over a wide range of participants, this study is the first one to estimate the reproducibility of PWV measurements that may be applicable in clinical practice, with the results being expressed using a simple measure of variability: a range of values.

We showed that depending on the number of repeated measurements used in the PWV estimation, the PWV repeatability ranged from unacceptable (a single measurement, two measurements, or two measurements with the inclusion of a third measurement as per the AHA guidance) to acceptable (four measurements). With there being only a slight increase in the proposed threshold for the minimal clinically important difference from 1 m/s (O’Connor, Koufaki et al., 2017) to 1.1 m/s, the four measurements represent an acceptable 11–14% of the participants with an intra-subject PWV range that is wider than the 1.1 m/s limit.

Our evaluation of the two precision components that are frequently confused in PWV research, the reproducibility and repeatability of PWV, showed that the former one is significantly larger than the latter one is, on average, by 0.7 m/s. It was also demonstrated that both of the devices exhibit an adequate repeatability PWV range according to the 1 m/s criteria.

We also investigated large discrepancies between the consecutive PWV measurements, which are defined by the AHA as discrepancies that are larger than 0.5 m/s, and we found they are prevalent with both of the devices. This fact, along with the seemingly random occurrence of large discrepancies per individual, demonstrates that identifying individuals with a significantly higher probability of large discrepancies is unlikely, and this supports the need for a new PWV estimation protocol. Instead of the sporadic inclusion of a third measurement when a discrepancy is large, the number of measurements should be increased for all of the participants from the current two measurements.

Finally, we identified several factors that influence the amount of variation between consecutive PWV measurements, with a clear difference between the devices in terms of the significant predictors detected. Age, MAP, the outdoor temperature, and the interaction between the outdoor temperature and MAP were all predictors for SphygmoCor. This implies that in the case of a low or high outdoor temperatures, the body’s adaptation to the room temperature in the lab may need to be extended beyond the standard 10 min resting time to minimize the differences between the consecutive measurements. For the Arteriograph, however, the predictors included personal characteristics such as BMI, sex, and hypertension status, which are less directly associated with arterial stiffness than SphygmoCor’s predictors are.

### 4.1. Number of Measurements Used in PWV Estimation

When one is translating the cfPWV research into clinical practice, the issue of the number of measurements used in the PWV evaluation is crucial, not only in the context of the desired precision of the PWV estimation, but also regarding time spent recording, as shorter procedure times would be required to ensure the proper workflow at the physician’s office. The evidence supporting the choice of the number of measurements used in PWV estimation, regardless of its importance, is scarce, and it currently relies on a single study [33]. Despite claiming that the reproducibility was assessed, the authors of this study, which included 80% men, took three PWV measurements that were taken using the Compilor device in a single recording session approximately 1 min apart and essentially assessed the effect of the number of measurements on PWV repeatability. Additionally, a poster by Souza et al. evaluated the impact of the number of measurements on the repeatability of PWV measurements taken in the elderly using the SphygmoCor device [45]. To our knowledge, this is the first study to investigate this effect in terms of PWV reproducibility, which were assessed here under a broad range of conditions and in a broad range of participants. Our results show that based on the effect on repeatability, we do not recommend using single or two measurements, including the procedure proposed by the current PWV guidance [22,46] to estimate the PWV. Currently, the optimal precision is observed when four measurements were taken, and the threshold of 1.1 m/s represents the minimal clinically important difference, but these results should be supported with future studies to increase the strength of the evidence and precisely balance the findings with a need to simplify the measurement techniques in clinics and utilize as few measures as possible to decrease the time spent testing.

### 4.2. PWV Repeatability and Reproducibility: Is There a Difference?

Except for the clinical change in PWV measurements due to underlying pathophysiological mechanism(s), the estimate of PWV reproducibility should account for as many sources of PWV variability as one might reasonably expect to encounter in clinical settings. Having said that, different observers or times of day may also be sources of variation in the data. However, in the context of estimating PWV repeatability, which is defined as the precision of PWV measurements obtained under the same conditions within 24 h, the intra-subject PWV variability that we recorded within 1 day might reflect a less conservative assessment of repeatability. Namely, this estimate might be inflated by additional variability due to different conditions such as different observers or different times of the day. Still, as was shown in our previous study, these sources (which involved the same amount of training for the observers) did not affect the PWV measurements, and they are unlikely to inflate the repeatability [41].

When PWV repeatability from our study was expressed as CV, it was consistent with the previously reported repeatability estimates for both the SphygmoCor [39] and the Arteriograph (Li, Cordes et al. 2014; Ring, Eriksson et al. 2014), including the Li Y et al. study, in which the measurements were taken at different times of the day. Furthermore, our finding, as well as the findings of other studies reporting PWV repeatability using CV, were consistent with the estimates of PWV reproducibility from the three randomized controlled studies that monitored the PWV changes over time [40]. The consistency of all of these CVs demonstrates the difficulty in estimating precision when a relative measure of variability is used for the estimation (CV, but also ICC, Pearson’s correlation coefficient, and others), and it may be one of the reasons why repeatability and reproducibility are often confused in studies [33,37,38,39,40]. Although some of these studies employed different observers, which may justify their use of the term reproducibility, their definition does not correspond to the AHA’s definition of PWV reproducibility for measurements that are taken more than 24 h apart.

Contrary to CV comparisons, when we compared the PWV repeatability and reproducibility using ranges of values estimated from two consecutive measurements, as is currently recommended by AHA, our results demonstrated a clinically important difference of 0.7 m/s between the PWV repeatability and reproducibility. Moreover, the PWV repeatability was good for both of the devices, as just 14% (SphygmoCor) and 13% (Arteriograph) of the recording days presented with a range of wider than ≤1 m/s for and just 10% of them had a range of wider than ≤1.1 m/s.

### 4.3. Discrepancies between Consecutive PWV Measurements

To the best of our knowledge, this is the first study in which discrepancies between the consecutive PWV measurements including the occurrence of large discrepancies (>0.5 m/s) and the size of the discrepancies were investigated in more detail. The results altogether point towards important differences between the devices.

We showed that the odds of observing one pair of measurements with a large discrepancy increases with the median value of a person’s MAP or DBP for SphygmoCor or HR for Arteriograph, but they are not affected by the experimental or outdoor meteorological conditions we tested or personal characteristics such as age, sex, BMI, or hypertensive status. At least part of the effect of these physiological variables is due to their natural variation within a person. The variability of the BPs increases with the average value of the BP, and it is positively associated with the severity of organ damage and cardiovascular morbidity and mortality in hypertensive patients and the general population [47]. The variability of the HR, on the other hand, results from complex, nonlinear interactions in a number of different physiological systems [48]. On average, both the MAP and DBP deviated within a person by 6 mmHg, whereas the HR deviated by 6 bpm with an intra-subject ranges from 7 to 32 mmHg (for MAP) and from 6 to 30 bpm (for HR). The difference between the devices, in terms of the physiological variables identified, which are significant predictors of a pair of measurements with a large discrepancy, is most likely due to their distinct modes of operation, with the applanation tonometry’s cfPWV seemingly providing a more direct estimate of the arterial stiffness due to its association with the BPs [1,49,50].

In line with the abovementioned results, we also identified factors that influence the size of the discrepancy between the consecutive PWV measurements, and we again found a distinct difference in terms of significant predictors detected between the devices. The findings of the SphygmoCor show that this variation is greater in older adults with stiffer arteries. Furthermore, we discovered a significant interaction between a person’s MAP and outdoor temperature, implying that the outdoor temperature has a moderating effect on the relationship between MAP and the amount of variation. This finding suggests that in the case of an externally low or high temperature, the body’s adaption to the room temperature in the lab may need to be extended beyond the typical 10 min resting time to minimize the differences between the consecutive measurements. Personal characteristics such as BMI, sex, and hypertension status affect the size of differences between the consecutive PWV measurements taken using the Arteriograph. However, these characteristics do not directly link to arterial stiffness, as is the case with the SphygmoCor, whose predictors included age, MAP, and temperature, nor can they be targeted to reduce the amount of variation.

### 4.4. On Variability in PWV in General

While the variability in the PWV can be an annoyance when one is attempting to determine the true PWV value, and while it affects the resolution of minimal clinically important differences in the PWV, the sensitivity of PWV measurements to the current status of the arterial tree, including the pulse pressure distension due to changes in the BPs, is critical if one wishes to monitor clinical PWV changes over time. In that context, while some devices may appear to be superior in terms of PWV repeatability and reproducibility to others [39], it is questionable how sensitive their mode of operation is to pathological changes. Only the clinical evaluation of these devices in terms of comparisons of the efficacy of therapies driven by PWV or with PWV as a therapeutic target can determine which device is best one to be used in the clinical context.

### 4.5. Limitations

The fact that individuals with hypertension were told to keep taking their vasoactive medication as directed throughout the study is one of the potential limitations of the study. The reason for such guidance was that we were interested in the intra-subject variability in the PWV measurements, rather than the absolute PWV levels. That being said, we anticipated that irregular compliance with vasoactive medication would have had a significantly greater impact on our results than taking vasoactive medication throughout the study would have had, as in the latter case the same effect of the medicine is expected on all of the measurements. In addition, in future clinical trials involving PWV, we expect that PWV levels will be evaluated in patients undergoing treatments. The same approach as ours was used in a recent study by Keehn L et al. [40] focusing on longitudinal changes.

The study did not assess the effect of changing therapies on the PWV reproducibility in patients, which could have occurred over a longer follow-up period. Drugs being introduced or changes in dosage after the baseline PWV measurement has been taken (e.g., sartans, which affect the arteries differently than calcium antagonists, statins, or diabetic therapies do) may affect this reproducibility [51,52]. Future studies should investigate these pharmacological effects.

We included measurements that were taken within 1 day but under different conditions—by different observers and at different times of day—when we were estimating the PWV repeatability, which may have inflated the repeatability estimate. However, as previously stated, these conditions did not affect the PWV measurements in our previous study (the observers received an equal amount of training), and we do not expect them to significantly affect the repeatability estimate [41].

## 5. Conclusions

We showed that the current AHA PWV estimation guidance is suboptimal as the PWV range was outside the 1 m/s threshold, which is a proposed minimal clinically important difference, for most of the participants. We yielded the best reproducibility with a median of for measurements and a 1.1 m/s threshold.

Regarding the PWV reproducibility and repeatability, which are frequently used interchangeably in studies, while the range showed the clinically relevant difference between them, the relative measures of variability were comparable.

We also found that different physiological variables were predictors of the discrepancy between the consecutive measurements, which points toward their distinct modes of operation. Age, MAP, the outdoor temperature, and the interaction between the outdoor temperature and MAP were all predictors for SphygmoCor. This implies that in the case of a low or high outdoor temperature, the body’s adaptation to the room temperature in the lab may need to be extended beyond the standard 10 min resting time to minimize the differences between the consecutive measurements. For the Arteriograph, however, the predictors included personal characteristics such as BMI, sex, and hypertension status, which are less directly associated with arterial stiffness than the SphygmoCor’s predictors are.

## Figures and Tables

**Table 1:** Predictors of the size of discrepancies between the two consecutive quality-passed PWV measurements.

Discrepancies		B	95% CI		*p*-Value
**SphygmoCor (cfPWV)**	**Age (years)**	0.01	0.004	0.02	0.003 *
	**Outdoor temperature (°C)**	0.25	0.05	0.44	0.015 *
	**MAP (mmHg)**	0.05	0.02	0.08	0.002 *
	**Interaction Outdoor** **temperature × MAP**	−0.003	−0.005	−0.0008	0.007 *
			
	ICC at a participant level 3.17 ×10^−14^ (95% CI 3.17 ×10^−14^–3.17 ×10^−14^), Snijders/Bosker R^2^ Level 1: 12%, Level 2: 21%				
**Arteriograph (PWVao)**	**BMI**	0.03	0.003	0.05	0.025 *
	**Sex (Female vs. Male)**	0.29	0.08	0.50	0.007 *
	**Hypertension (Yes vs. No)**	−0.23	−0.44	−0.01	0.037 *
	ICC at a participant level 0.11 (95% CI 0.05–0.21), Snijders/Bosker R^2^ Level 1: 10%, Level 2: 30%				

The models used are the mixed effects ML regression models with a robust estimator. * Significant at the 0.05 level.

## Data Availability

The data presented in this study are available on request from the corresponding author.

## References

[1] Nichols W.W., McDonald D.A. (2011). McDonald’s Blood Flow in Arteries: Theoretic, Experimental, and Clinical Principles.

[2] Laurent S., Boutouyrie P., Cunha P.G., Lacolley P., Nilsson P.M. (2019). Concept of Extremes in Vascular Aging. Hypertension.

[3] Ben-Shlomo Y., Spears M., Boustred C., May M., Anderson S.G., Benjamin E.J., Boutouyrie P., Cameron J., Chen C.H., Cruickshank J.K. (2014). Aortic pulse wave velocity improves cardiovascular event prediction: An individual participant meta-analysis of prospective observational data from 17,635 subjects. J. Am. Coll. Cardiol..

[4] Vlachopoulos C., Aznaouridis K., Stefanadis C. (2010). Prediction of cardiovascular events and all-cause mortality with arterial stiffness: A systematic review and meta-analysis. J. Am. Coll. Cardiol..

[5] Zhong Q., Hu M.J., Cui Y.J., Liang L., Zhou M.M., Yang Y.W., Huang F. (2018). Carotid-Femoral Pulse Wave Velocity in the Prediction of Cardiovascular Events and Mortality: An Updated Systematic Review and Meta-Analysis. Angiology.

[6] Boutouyrie P., Chowienczyk P., Humphrey J.D., Mitchell G.F. (2021). Arterial Stiffness and Cardiovascular Risk in Hypertension. Circ. Res..

[7] Chirinos J.A., Segers P., Hughes T., Townsend R. (2019). Large-Artery Stiffness in Health and Disease: JACC State-of-the-Art Review. J. Am. Coll. Cardiol..

[8] Nilsson P.M., Boutouyrie P., Laurent S. (2009). Vascular aging: A tale of EVA and ADAM in cardiovascular risk assessment and prevention. Hypertension.

[9] Laurent S. (2005). Arterial stiffness: Intermediate or surrogate endpoint for cardiovascular events?. Eur. Heart J..

[10] Della Corte V., Tuttolomondo A., Pecoraro R., Di Raimondo D., Vassallo V., Pinto A. (2016). Inflammation, Endothelial Dysfunction and Arterial Stiffness as Therapeutic Targets in Cardiovascular Medicine. Curr. Pharm. Des..

[11] Mattace-Raso F.U., van der Cammen T.J., Hofman A., van Popele N.M., Bos M.L., Schalekamp M.A., Asmar R., Reneman R.S., Hoeks A.P., Breteler M.M. (2006). Arterial stiffness and risk of coronary heart disease and stroke: The Rotterdam Study. Circulation.

[12] Mitchell G.F., Hwang S.J., Vasan R.S., Larson M.G., Pencina M.J., Hamburg N.M., Vita J.A., Levy D., Benjamin E.J. (2010). Arterial stiffness and cardiovascular events: The Framingham Heart Study. Circulation.

[13] Sehestedt T., Jeppesen J., Hansen T.W., Rasmussen S., Wachtell K., Ibsen H., Torp-Pedersen C., Olsen M.H. (2009). Risk stratification with the risk chart from the European Society of Hypertension compared with SCORE in the general population. J. Hypertens..

[14] Kaess B.M., Rong J., Larson M.G., Hamburg N.M., Vita J.A., Cheng S., Aragam J., Levy D., Benjamin E.J., Vasan R.S. (2016). Relations of Central Hemodynamics and Aortic Stiffness with Left Ventricular Structure and Function: The Framingham Heart Study. J. Am. Heart Assoc..

[15] Kollias A., Lagou S., Zeniodi M.E., Boubouchairopoulou N., Stergiou G.S. (2016). Association of Central Versus Brachial Blood Pressure With Target-Organ Damage: Systematic Review and Meta-Analysis. Hypertension.

[16] Lu Y., Zhu M., Bai B., Chi C., Yu S., Teliewubai J., Xu H., Wang K., Xiong J., Zhou Y. (2017). Comparison of Carotid-Femoral and Brachial-Ankle Pulse-Wave Velocity in Association With Target Organ Damage in the Community-Dwelling Elderly Chinese: The Northern Shanghai Study. J. Am. Heart Assoc..

[17] Vasan R.S., Short M.I., Niiranen T.J., Xanthakis V., DeCarli C., Cheng S., Seshadri S., Mitchell G.F. (2019). Interrelations Between Arterial Stiffness, Target Organ Damage, and Cardiovascular Disease Outcomes. J. Am. Heart Assoc..

[18] Upadhya B., Pajewski N.M., Rocco M.V., Hundley W.G., Aurigemma G., Hamilton C.A., Bates J.T., He J., Chen J., Chonchol M. (2021). Effect of Intensive Blood Pressure Control on Aortic Stiffness in the SPRINT-HEART. Hypertension.

[19] Laurent S., Chatellier G., Azizi M., Calvet D., Choukroun G., Danchin N., Delsart P., Girerd X., Gosse P., Khettab H. (2021). SPARTE Study: Normalization of Arterial Stiffness and Cardiovascular Events in Patients With Hypertension at Medium to Very High Risk. Hypertension.

[20] Laurent S., Cockcroft J., Van Bortel L., Boutouyrie P., Giannattasio C., Hayoz D., Pannier B., Vlachopoulos C., Wilkinson I., Struijker-Boudier H. (2006). Expert consensus document on arterial stiffness: Methodological issues and clinical applications. Eur. Heart J..

[21] Cooke A.B., Kuate Defo A., Dasgupta K., Papaioannou T.G., Lee J., Morin S.N., Murphy J., Santosa S., Daskalopoulou S.S. (2021). Methodological considerations for the measurement of arterial stiffness using applanation tonometry. J. Hypertens..

[22] Townsend R.R., Wilkinson I.B., Schiffrin E.L., Avolio A.P., Chirinos J.A., Cockcroft J.R., Heffernan K.S., Lakatta E.G., McEniery C.M., Mitchell G.F. (2015). Recommendations for Improving and Standardizing Vascular Research on Arterial Stiffness: A Scientific Statement From the American Heart Association. Hypertension.

[23] Cooke A.B., Ta V., Iqbal S., Gomez Y.H., Mavrakanas T., Barre P., Vasilevsky M., Rahme E., Daskalopoulou S.S. (2018). The Impact of Intradialytic Pedaling Exercise on Arterial Stiffness: A Pilot Randomized Controlled Trial in a Hemodialysis Population. Am. J. Hypertens..

[24] Dasgupta K., Rosenberg E., Daskalopoulou S.S. (2014). Step Monitoring to improve ARTERial health (SMARTER) through step count prescription in type 2 diabetes and hypertension: Trial design and methods. Cardiovasc. Diabetol..

[25] Doonan R.J., Mutter A., Egiziano G., Gomez Y.H., Daskalopoulou S.S. (2013). Differences in arterial stiffness at rest and after acute exercise between young men and women. Hypertens. Res..

[26] Doonan R.J., Scheffler P., Yu A., Egiziano G., Mutter A., Bacon S., Carli F., Daskalopoulos M.E., Daskalopoulou S.S. (2011). Altered arterial stiffness and subendocardial viability ratio in young healthy light smokers after acute exercise. PLoS ONE.

[27] Karamat F., Diemer F., Van Montfrans G., Oehlers G., Brewster L. (2016). PS 09-03 Arterial Stiffness in a Random Sample of a Multi-Ethnic Population in Suriname: The Helisur Study. J. Hypertens..

[28] Karpettas N., Destounis A., Kollias A., Nasothimiou E., Moyssakis I., Stergiou G.S. (2014). Prediction of treatment-induced changes in target-organ damage using changes in clinic, home and ambulatory blood pressure. Hypertens. Res..

[29] Harvey R.E., Barnes J.N., Hart E.C., Nicholson W.T., Joyner M.J., Casey D.P. (2017). Influence of sympathetic nerve activity on aortic hemodynamics and pulse wave velocity in women. Am. J. Physiol. Heart Circ. Physiol..

[30] Meani P., Maloberti A., Sormani P., Colombo G., Giupponi L., Stucchi M., Varrenti M., Vallerio P., Facchetti R., Grassi G. (2018). Determinants of carotid-femoral pulse wave velocity progression in hypertensive patients over a 3.7 years follow-up. Blood Press..

[31] Hudson L.D., Kinra S., Wong I.C.K., Viner R.M. (2017). Arterial stiffening, insulin resistance and acanthosis nigricans in a community sample of adolescents with obesity. Int. J. Obes..

[32] Salvi P., Furlanis G., Grillo A., Pini A., Salvi L., Marelli S., Rovina M., Moretti F., Gaetano R., Pintassilgo I. (2019). Unreliable Estimation of Aortic Pulse Wave Velocity Provided by the Mobil-O-Graph Algorithm-Based System in Marfan Syndrome. J. Am. Heart Assoc..

[33] Papaioannou T.G., Protogerou A.D., Nasothimiou E.G., Tzamouranis D., Skliros N., Achimastos A., Papadogiannis D., Stefanadis C.I. (2012). Assessment of differences between repeated pulse wave velocity measurements in terms of ‘bias’ in the extrapolated cardiovascular risk and the classification of aortic stiffness: Is a single PWV measurement enough?. J. Hum. Hypertens..

[34] Milan A., Zocaro G., Leone D., Tosello F., Buraioli I., Schiavone D., Veglio F. (2019). Current assessment of pulse wave velocity: Comprehensive review of validation studies. J. Hypertens..

[35] Tripkovic L., Hart K.H., Frost G.S., Lodge J.K. (2014). Interindividual and intraindividual variation in pulse wave velocity measurements in a male population. Blood Press. Monit..

[36] Kallem R.R., Meyers K.E.C., Sawinski D.L., Townsend R.R. (2013). Variation and variability in carotid-femoral pulse wave velocity. Artery Res..

[37] Laugesen E., Rossen N.B., Hoyem P., Christiansen J.S., Knudsen S.T., Hansen K.W., Hansen T.K., Poulsen P.L. (2013). Reproducibility of pulse wave analysis and pulse wave velocity in patients with type 2 diabetes. Scand. J. Clin. Lab. Investig..

[38] Lee N.B., Park C.G. (2009). Reproducibility of regional pulse wave velocity in healthy subjects. Korean J. Intern. Med..

[39] Grillo A., Parati G., Rovina M., Moretti F., Salvi L., Gao L., Baldi C., Sorropago G., Faini A., Millasseau S.C. (2017). Short-Term Repeatability of Noninvasive Aortic Pulse Wave Velocity Assessment: Comparison Between Methods and Devices. Am. J. Hypertens..

[40] Keehn L., Hall W.L., Berry S.E., Sanders T.A.B., Chowienczyk P., Floyd C.N. (2022). Reproducibility of sequential ambulatory blood pressure and pulse wave velocity measurements in normotensive and hypertensive individuals. J. Hypertens..

[41] Podrug M., Šunjić B., Bekavac A., Koren P., Đogaš V., Mudnić I., Boban M., Jerončić A. (2023). The effects of experimental, meteorological, and physiological factors on short-term repeated pulse wave velocity measurements, and measurement difficulties: A randomized crossover study with two devices. Front. Cardiovasc. Med..

[42] Dhand N.K., Khatkar M.S. Statulator: An online statistical calculator. Sample Size Calculator for Estimating a Single Mean. http://statulator.com/SampleSize/ss1M.html.

[43] Field A.P., Wilcox R.R. (2017). Robust statistical methods: A primer for clinical psychology and experimental psychopathology researchers. Behav. Res. Ther..

[44] White H. (1980). A heteroskedasticity-consistent covariance matrix estimator and a direct test for heteroskedasticity. Econometrica.

[45] Souza D.F., Brunelli A.C.D., Peres C.I., Dorneles M.C., Nolasco G.D., Mendonca G.S., Freitas E.G., Peixoto A.J., Ferreira S.R. (2016). Agreement Among Sequential Carotid-Femoral Pulse Wave Velocity (Cf-Pwv) Measurements In Elderly Hypertensive Patients((star)). J. Hypertens..

[46] Wilkinson I.B., McEniery C.M., Schillaci G., Boutouyrie P., Segers P., Donald A., Chowienczyk P.J. (2010). ARTERY Society guidelines for validation of non-invasive haemodynamic measurement devices: Part 1, arterial pulse wave velocity. Artery Res..

[47] Parati G., Torlasco C., Pengo M., Bilo G., Ochoa J.E. (2020). Blood pressure variability: Its relevance for cardiovascular homeostasis and cardiovascular diseases. Hypertens. Res..

[48] McCraty R., Shaffer F. (2015). Heart Rate Variability: New Perspectives on Physiological Mechanisms, Assessment of Self-regulatory Capacity, and Health Risk. Glob. Adv. Health Med..

[49] Giannattasio C., Failla M., Mangoni A.A., Scandola L., Fraschini N., Mancia G. (1996). Evaluation of arterial compliance in humans. Clin. Exp. Hypertens..

[50] Kim E.J., Park C.G., Park J.S., Suh S.Y., Choi C.U., Kim J.W., Kim S.H., Lim H.E., Rha S.W., Seo H.S. (2007). Relationship between blood pressure parameters and pulse wave velocity in normotensive and hypertensive subjects: Invasive study. J. Hum. Hypertens..

[51] Peng F., Pan H., Wang B., Lin J., Niu W. (2015). The impact of angiotensin receptor blockers on arterial stiffness: A meta-analysis. Hypertens. Res..

[52] Lo Gullo A., Giuffrida C., Morace C., Squadrito G., Magnano San Lio P., Ricciardi L., Salvarani C., Mandraffino G. (2022). Arterial Stiffness and Adult Onset Vasculitis: A Systematic Review. Front. Med..

